# A review of the Nearctic genus *Prostoia* (Ricker) (Plecoptera, Nemouridae), with the description of a new species and a surprising range extension for *P. hallasi* Kondratieff & Kirchner

**DOI:** 10.3897/zookeys.401.7299

**Published:** 2014-04-14

**Authors:** Scott A. Grubbs, Richard W. Baumann, R. Edward DeWalt, Tari Tweddale

**Affiliations:** 1 Department of Biology and Center for Biodiversity Studies, Western Kentucky University, Bowling Green, Kentucky, 42101, USA; 2 Department of Biology and Monte L. Bean Life Science Museum, Brigham Young University, Provo, Utah, 84602, USA; 3 University of Illinois, Prairie Research Institute, Illinois Natural History Survey, 1816 S Oak St., Champaign, Illinois, 61820, USA

**Keywords:** Plecoptera, Nemouridae, *Prostoia*, new species, North America

## Abstract

The Nearctic genus *Prostoia* (Plecoptera: Nemouridae) is reviewed. *Prostoia ozarkensis* **sp. n.** is described from the male and female adult stages mainly from the Interior Highland region encompassing portions of Arkansas, Missouri, and Oklahoma. *Prostoia ozarkensis* **sp. n.** appears most closely related to two species, one distributed broadly across the western Nearctic region, *P. besametsa* (Ricker), and one found widely throughout the central and eastern Nearctic regions, *P. completa* (Walker). A surprising range extension is noted for *P. hallasi* Kondratieff & Kirchner, a species once known only from the Great Dismal Swamp, from small upland streams in southern Illinois. Additional new state records are documented for *P. besametsa*, *P. completa*, *P. hallasi* and *P. similis* (Hagen). Taxonomic keys to *Prostoia* males and females are provided, and scanning electron micrographs of adult genitalia of all species are given.

## Introduction

*Prostoia* Ricker (Plecoptera: Nemouridae) was erected as a subgenus to include three species: *Nemoura (Prostoia) besametsa* Ricker, 1952, *Nemoura (Prostoia) completa* Walker, 1852, and *Nemoura (Prostoia) similis* Hagen, 1861 (Ricker 1952). *Prostoia* was later raised to full generic rank by Illies (1966). A fourth species, *Prostoia hallasi*, was described by Kondratieff and Kirchner (1984).

*Prostoia besametsa* is the sole species found in the western Nearctic region, distributed broadly from California east to New Mexico and north to Alaska (Baumann et al. 1977, Stewart and Oswood 2006, DeWalt et al. 2013). *Prostoia completa* and *Prostoia similis* are both found widely throughout the central and eastern Nearctic regions (DeWalt et al. 2013). *Prostoia hallasi*, in contrast, has been reported only from the Great Dismal Swamp (Kondratieff and Kirchner 1984, Kondratieff et al. 1995), an Atlantic Coastal wetland located in North Carolina and Virginia (Traylor 2010).

*Prostoia* males are easily identified among Nemouridae by their simple, elongate, anteriorly-recurved epiproct that is comprised almost entirely by the ventral sclerite (Baumann 1975). The dorsal sclerite is reduced to a pair of lateral arms located along each side of the epiproct base, except in *Prostoia hallasi* where they are secondarily absent (Ricker 1952, Kondratieff and Kirchner 1984). Wing coloration ranges from uniformly dark brown in *Prostoia hallasi* to mottled with a distinctive light band near the apex in all other species.

For several years, Bill P. Stark (Mississippi College, Clinton, Mississippi) and the second author suspected that *Prostoia completa* from the Ozark Plateau region of northern Arkansas, southern Missouri, and eastern Oklahoma represented an undescribed species. The new species is described herein, with brief anecdotes and new state records of the four previously-described species. Species keys to the male and female adult stages are provided.

## Materials and methods

*Prostoia* specimens used in this study were obtained from, or deposited in, the following collections: B.P. Stark Collection, Mississippi College, Clinton (BPSC); Monte L. Bean Museum, Brigham Young University, Provo, Utah (BYUC); Canadian National Collection of Insects, Ottawa (CNCI); Colorado State University Collection, Fort Collins (CSUC); University of Guelph Insect Collection, Guelph (DEBU); Illinois Natural History Survey, Champaign-Urbana (INHS); Michigan State University Arthropod Research Collection, East Lansing (MSUC); Ohio Environmental Protection Agency, Groveport (OEPA); Purdue University Research Collection, West Lafayette, Indiana (PURC); R.F. Kirchner Personal Collection, Huntington, West Virginia (RFKC); Royal Ontario Museum, Toronto (ROME); University of Michigan Museum of Zoology Insect Collection, Ann Arbor (UMMZ); University of Minnesota Insect Collection, St. Paul (UMSP); University of Notre Dame Insect Collection, South Bend, Indiana (UNDIC); United States National Museum Collection, Smithsonian Institution, Washington, D.C. (USNM); University of Wisconsin Entomological Research Center, Madison (UWIRC); and the S.A. Grubbs Collection, Western Kentucky University, Bowling Green (WKUC).

All specimen records for *Prostoia hallasi* and the new species were included herein. Due to the large volume of material examined for *Prostoia besametsa*, *Prostoia completa*, and *Prostoia similis*, however, these data are available only in a corresponding appendix (see Suppl. material 1 at end of paper).

Locality data, in decimal degrees, for each specimen record were obtained either directly with hand-held GPS units on site or georeferenced from museum label data (if possible). Specimens were studied with scanning electron microscopy (SEM) with a Philips XL30 ESEM FEG electron microscope at Brigham Young University.

## Results and discussion

### Key to the *Prostoia* adults

**Table:** 

1	Male	2
–	Females	6
2	Dorsal sclerite lacks lateral arms (Figs 17–18, 22–23); anterior terminus of ventral sclerite bears an ornate, secondarily divided process apically (Figs 18–21)	*Prostoia hallasi* Kondratieff & Kirchner
–	Dorsal sclerite possesses lateral arms (Figs 3, 5, 11, 13, 28–29, 34, 37); anterior terminus of ventral sclerite simple, not divided apically (Figs 2, 4, 10, 12, 26, 28, 34, 36)	3
3	Lateral arms long and sinuate, reaching ca. ½ the length of the ventral sclerite (Figs 33–34, 37–38)	*Prostoia similis* (Hagen)
–	Lateral arms compact and markedly shorter, extending <¼ the length of the ventral sclerite (Figs 3, 5, 11, 13, 28–29)	4
4	In dorsal view, anterior terminus of ventral sclerite narrowing gradually to a V-shaped tip, subterminal portion markedly wider than posterior portion (Figs 1–2); lateral arms highly-reduced and can be difficult to see with light microscopy, not extending beyond base of dorsal sclerite (Figs 3, 5); widespread western Nearctic species	*Prostoia besametsa* (Ricker)
–	In dorsal view, anterior terminus of ventral sclerite set apart from majority of sclerite, tip near parallel-sided, subterminal portion only slightly wider than posterior portion (Figs 9–10, 25–26); lateral arms extending beyond base of dorsal sclerite (Figs 11, 13, 15, 27, 29, 31); central and eastern Nearctic species	5
5	In dorsal view, anterior portion of ventral sclerite parallel-sided beyond recurved base (Figs 9–10); in lateral view, posterior portion of ventral sclerite only slightly deflected ventrally (Fig. 11); tip of ventral sclerite slightly deflected upward, parallel-sided and subquadrate apically (Figs 9, 12, 14); lateral arms sickle-shaped, gradually recurved (Fig. 15)	*Prostoia completa* (Walker)
–	In dorsal view, anterior portion of ventral sclerite not parallel-sided, gradually expanding laterally beyond recurved base (Figs 25–27); in lateral view, posterior portion of ventral sclerite deflected ventrally (Fig. 28); tip of ventral sclerite not deflected upward (Figs 28–30); lateral arms triangular in shape, tips flared laterally (Figs 29, 31)	*Prostoia ozarkensis* Baumann & Grubbs, sp. n.
6	The 7^th^ and 8^th^ abdominal sterna not fused medially, with a well-developed subgenital plate that is convex, extending over the anteromedial margin of the 9^th^ sternum and very slightly notched medially (Fig. 24)	*Prostoia hallasi* Kondratieff & Kirchner
–	The 7^th^ and 8^th^ abdominal sterna fused medially, subgenital plate not convex and bearing a distinct medial notch (Figs 39–40) or not (Figs 8, 16, 32)	7
7	Posterior margin of 8^th^ sternum with a distinct medial notch, lateral lobes of subgenital plate distinctly angular, projecting ca. 1/4^th^ over anterior margin of 9^th^ sternum (Figs 39–40)	*Prostoia similis* Hagen
–	Posterior margin of 8^th^ sternum with, at best, a shallow medial notch, lateral lobes either projecting posteriorly or not (Figs 8, 16, 32)	8
8	Posterior margin of 8^th^ sternum essentially straight, lateral lobes not projecting posteriorly as a subgenital plate (Figs 7–8; Baumann et al. 1977, Fig. 107); widespread western Nearctic species	*Prostoia besametsa* (Ricker)
–	Posterior margin of 8^th^ sternum with a shallow median notch, lateral lobes broadly rounded, extending < 1/4^th^ over anterior margin of 9^th^ sternum (Figs 16, 32); central or eastern Nearctic species	9
9	Subgenital plate as in Fig. 16; eastern and central Nearctic species, known from eastern Canada south to Alabama and Mississippi, extending westward only to Iowa and Minnesota (Fig. 41)	*Prostoia completa* (Walker)
–	Subgenital plate as in Fig. 32; central Nearctic species, known from Shawnee Hills region of southern Illinois west to the Ozark Plateau region encompassing southern Missouri, northern Arkansas, and eastern Oklahoma (Fig. 42)	*Prostoia ozarkensis* Baumann & Grubbs, sp. n.

### Prostoia besametsa

(Ricker)

http://species-id.net/wiki/Prostoia_besametsa

http://lsid.speciesfile.org/urn:lsid:Plecoptera.speciesfile.org:TaxonName:6099

Figs 1–8 , 41

Nemoura (Prostoia) besametsa Ricker, 1952: 48. Holotype ♂ (INHS), Vedder Crossing, British Columbia, Canada
Nemoura glabra (in part) Claassen, 1923: 281
Nemoura glabra (in part) Needham & Claassen, 1925: 202. Syn. Illies, 1966: 221
Prostoia besametsa : Illies 1966: 220
Prostoia besametsa : Zwick 1973: 345
Prostoia besametsa : Baumann 1975: 27
Prostoia besametsa : Baumann et al. 1977: 38

#### Material examined

(Suppl. material 1).

#### Distribution.

Canada: AB, BC (DeWalt et al. 2013), NT (Stewart and Oswood 2006), YK (Stewart and Ricker 1997); USA: AK (Stewart and Oswood 2006), CA, CO, ID, MT, NM, NV, OR, SD, UT, WA, WY (DeWalt et al. 2013), NE (New state record).

#### Remarks.

*Prostoia besametsa*, *Prostoia completa* and *Prostoia ozarkensis* sp. n. appear to form a species group based on structural similarities of the male ventral sclerite and lateral arms of the dorsal sclerite, and the female subgenital plate. The Black Hills region of eastern Wyoming and western South Dakota, plus the Sand Hills region of northwestern Nebraska, mark the eastern edge of this widespread western Nearctic species in the USA (Fig. 41), and well distant from the closest distribution point of *Prostoia completa* (Fig. 41) and *Prostoia ozarkensis* sp. n. (Fig. 42) (Huntsman et al. 1999, DeWalt et al. 2013). *Prostoia besametsa* is typically found in greatest numbers in large streams and small rivers.

**Figures 1–8.**
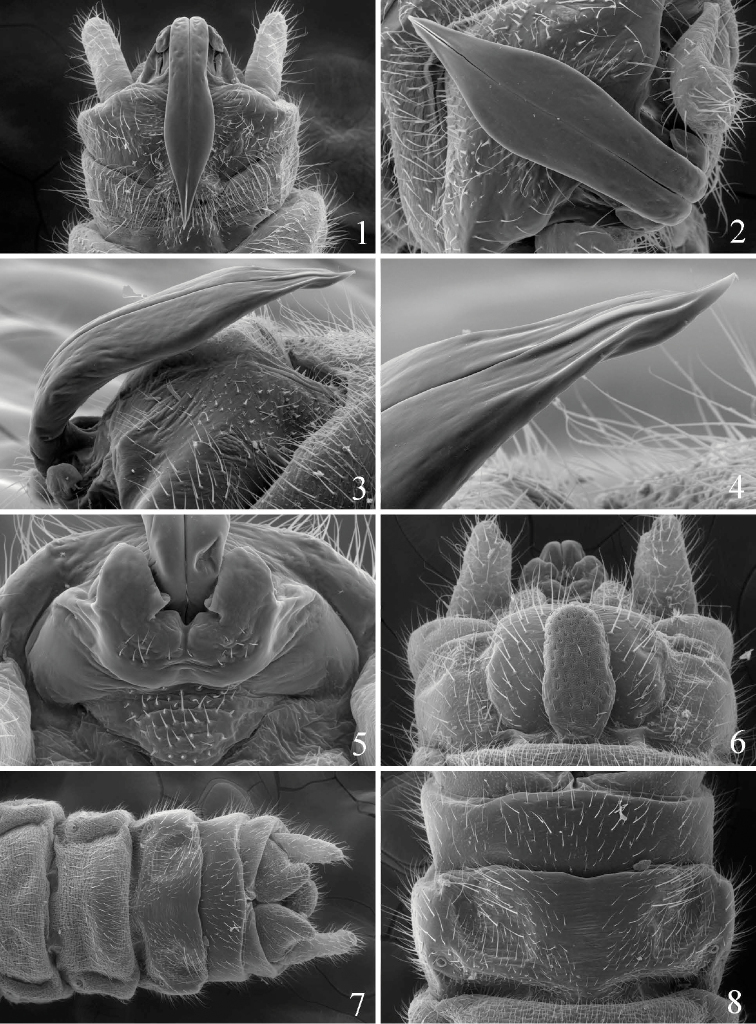
*Prostoia besametsa*, scanning electron micrographs, **1** USA, Utah, Monroe Creek, male, epiproct, dorsal view **2** USA, Montana, Gallatin River, male, epiproct, dorsal view **3** USA, South Dakota, Iron Creek, male, epiproct, lateral view **4** USA, South Dakota, Iron Creek, male, epiproct, lateral view **5** USA, Utah, Monroe Creek, male, abdominal terminalia, caudal view **6** USA, South Dakota, Iron Creek, male, abdominal terminalia, ventral view **7** USA, South Dakota, Iron Creek, female, abdominal terminalia, ventral view **8** USA, South Dakota, Iron Creek, female, abdominal terminalia, ventral view.

### Prostoia completa

(Walker)

http://species-id.net/wiki/Prostoia_completa

http://lsid.speciesfile.org/urn:lsid:Plecoptera.speciesfile.org:TaxonName:6101

Figs 9–16 , 41

Nemoura completa Walker, 1852: 191. Holotype ♂ (British Museum of Natural History, London), Nova Scotia, Canada
Nemoura glabra (in part) Claassen, 1923: 281. Syn. Illies, 1966: 221
Nemoura glabra : (in part) Needham & Claassen, 1925: 202.
Nemoura completa : Ricker 1938
Nemoura (Prostoia) completa : Ricker 1952: 49
Prostoia completa : Illies 1966: 221
Prostoia completa : Zwick 1973: 346
Prostoia completa : Baumann 1975: 27
Prostoia completa : Poulton and Stewart 1991: 29

#### Material examined

(Suppl. material 1).

#### Distribution.

Canada: NB, NS, ON, PE, PQ (DeWalt et al. 2013), NF (New provincial record); USA: AL, DE, IA, IN, KY, MA, ME, MI, MN, MS, NC, OH, PA, SC, TN, VA, WI, WV (DeWalt et al. 2013), MD (Grubbs 1997), NY (Myers et al. 2011), TN (New state record).

#### Remarks.

This species is distributed from Atlantic Canada to South Carolina and westward to Minnesota and Iowa (Fig. 41). Characteristics of the male epiproct remain constant from eastern Canada to the southeastern United States, without any indication of a north-south cline. However, populations from the northern Midwest are somewhat variable. The prior records of *Prostoia completa* from the Interior Highland region, namely the Ozark Plateau region (e.g. Poulton and Stewart 1991), now likely refer only to *Prostoia ozarkensis* sp. n., but very few specimens were available for this study. Specimens collected sporadically from the only locality in southern Illinois (Webb 2002, DeWalt and Grubbs 2011) were reexamined and now are considered *Prostoia ozarkensis* sp. n. This species was recently listed in Illinois as endangered due to it occurring in a single location in the state (Illinois Endangered Species Protection Board 2011). *Prostoia ozarkensis* sp. n. is very closely related to *Prostoia completa* and separable only by experts as this time. Examination of the relatedness of these two species and congeners using genetic markers is warranted given the implications for conservation status within Illinois.

Although the ranges of *Prostoia completa* and *Prostoia similis* (Fig. 42) overlap extensively throughout the eastern Nearctic region, the former species is typically associated with large streams and small rivers. *Prostoia completa* is less commonly collected from upland, headwater streams, except in the northeastern Nearctic region where both species sometimes occur at the same locality.

**Figures 9–16.**
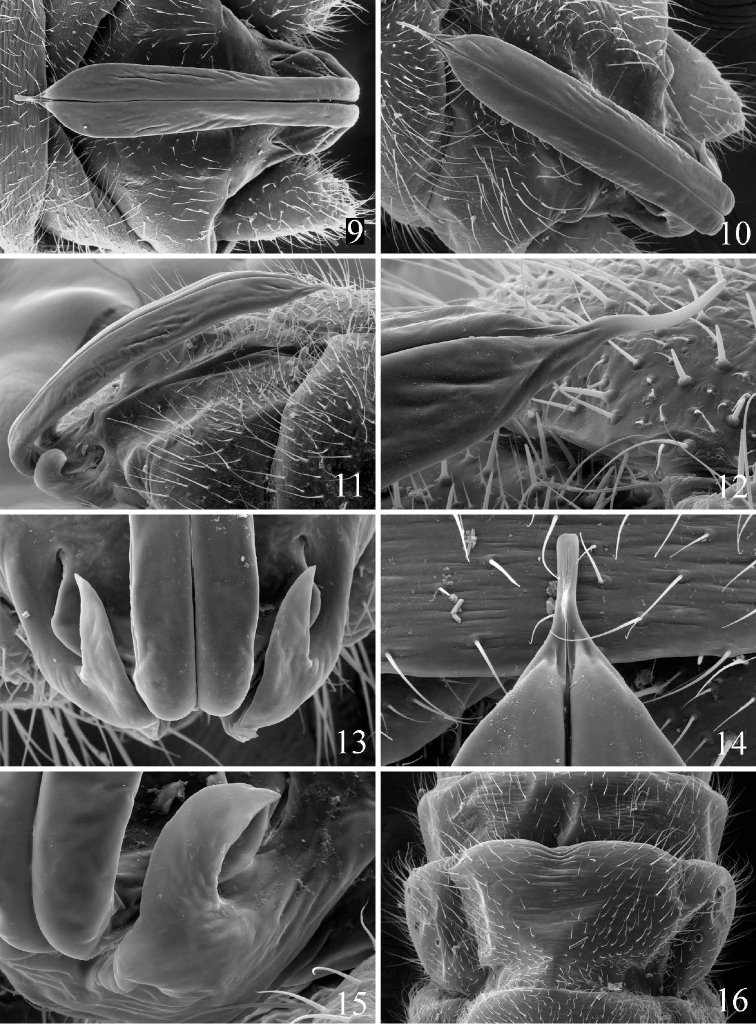
*Prostoia completa*, scanning electron micrographs, **9** USA, Wisconsin, Plover River, male, epiproct, dorsal view **10** Canada, Newfoundland, Walsh River, male, epiproct, dorsal view **11** Canada, Newfoundland, Walsh River, male, epiproct, lateral view **12** Canada, Newfoundland, Walsh River, male, epiproct tip, lateral view **13** USA, Virginia, Roanoke River, male, epiproct base, dorsal view **14** USA, Wisconsin, Plover River, male, epiproct tip, dorsal view **15** Canada, Newfoundland, Walsh River, male, epiproct base, lateral view **16** Canada, Newfoundland, Walsh River, female, abdominal terminalia, ventral view.

### Prostoia hallasi

Kondratieff & Kirchner

http://species-id.net/wiki/Prostoia_hallasi

http://lsid.speciesfile.org/urn:lsid:Plecoptera.speciesfile.org:TaxonName:6098

Figs 17–24 , 42

Prostoia hallasi Kondratieff & Kirchner, 1984: 579. Holotype ♂ (USNM), Washington Ditch, City of Suffolk, Virginia

#### Material examined

(also provided in Suppl. material 1). **USA**, **Connecticut**, New Haven Co., Hammonasset River, Killingworth, 41.3573, -72.6126, 1 May 1988, W.G. Downs, 3♂, 25♀ (BYUC). **Georgia**, Crisp Co., Gum Creek, Hwy 257, 32.0066, -83.7374, 30 March 1993, B.A. Caldwell, 2♂, 2♀, 2 nymphs (BYUC). **Illinois**, Pope Co., tributary to Alcorn Creek, 7.1 km NW Hamletsburg, 37.1777, -88.4953, 2 March 2012, R. E. DeWalt, 2♂, 2♀ (INHS), tributary to Alcorn Creek, 15 km NE Brookport, 37.1777, -88.4891, 17 March 2013 (reared, from nymphs collected 14 March 2013), S.A. Grubbs & J.M. Yates, 2♂, 2♀, 4 nymphs (WKUC), same site, 3 April 2013, S.A. Grubbs & J.M. Yates, 2♀ (WKUC). **Massachusetts**, Unknown County, “Boston Reg.”, 2 May 1936, L.J. Milne, 3♀ (USNM). **Virginia**, Essex Co., 1 mi SE Dunnsville, 37.8504, -76.8083 (malaise trap), 17–29 April 1992, D.R. Smith, 4♂, 110♀ (BYUC, USNM); same site, 26 March–8 April 1994, D.R. Smith, 4♂, 9♀ (BYUC, USNM); Falls Church City, Falls Church, 11 February 1941, J.F. Hanson, ♂ (USNM); Southhampton Co., Tarrara Creek, Hwy 666, 36.5952, -77.2274, 10 March 1991, R.W. Baumann & R.F. Kirchner, 2♂ (BYUC); Suffolk City, Washington Ditch, off Washington Ditch Road, Dismal Swamp, 36.6442, -76.5471, 2 March 1983, B.C. Kondratieff, 2♂, ♀ (paratypes; BYUC); Washington Ditch, Dismal Swamp, 36.6442, -76.5471, 10 March 1991, R.W. Baumann & R.F. Kirchner, 48♂, 54♀ (BYUC).

#### Distribution.

USA: NC, VA (DeWalt et al. 2013), CT, GA, IL, MA (New state records).

#### Remarks.

This species was once considered unique amongst Nearctic Nemouridae in that it was known only from low gradient coastal streams in the Great Dismal Swamp (Kondratieff and Kirchner 1984, Kondratieff et al. 1995). The discovery of localities north in New England and south to Georgia was not too surprising since these are range extensions along the Atlantic Coastal Plain (Fig. 42). This species should eventually be found in coastal regions within the intervening states (i.e. Delaware, Maryland, New Jersey, New York, Pennsylvania, and South Carolina).

**Figures 17–24.**
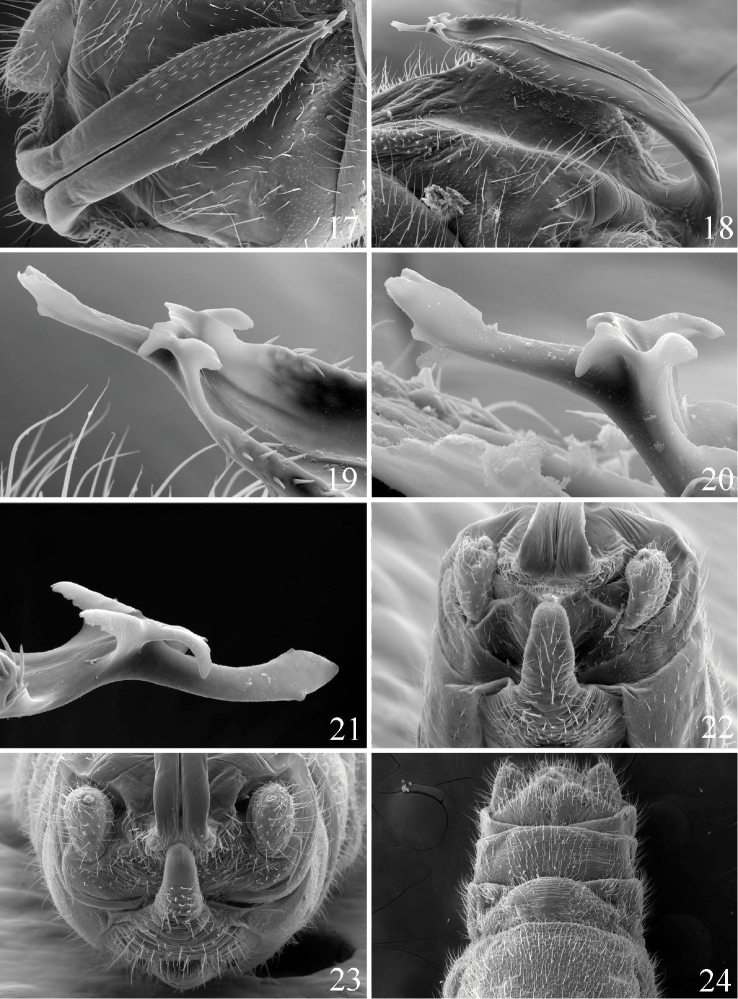
*Prostoia hallasi*, scanning electron micrographs, **17** USA, Virginia, Dunnsville, male, epiproct, dorsal view **18** USA, Virginia, Washington Ditch, male, epiproct, lateral view **19** USA, Virginia, Washington Ditch, male, epiproct tip, lateral view **20** USA, Connecticut, Hammonasset River, male, epiproct tip, lateral view **21** USA, Illinois, tributary to Alcorn Creek, male, epiproct tip, lateral view **22** USA, Illinois, tributary to Alcorn Creek, male, abdominal terminalia, ventral view **23** USA, Virginia, Washington Ditch, male, abdominal terminalia, caudal view **24** USA, Virginia, Dunnsville, female, abdominal terminalia, ventral view.

We initially anticipated that the populations from the Shawnee Hills region of southern Illinois represented an undescribed species. Both Illinois sites were small, upland tributaries ca. 1 m wide and very distinct from the description of the type locality (Kondratieff and Kirchner 1984). Yet the SEM images of the epiproct from specimens from several locations, particularly of the complex ornamentation of the terminus of the ventral sclerite, showed unexpected across-site similarity and no evidence that the southern Illinois populations represented an undescribed species. The epiproct terminus of the populations from Essex Co., Virginia (Fig. 17), coastal Connecticut (Fig. 20), southern Illinois (Fig. 21) and the type locality in eastern Virginia (Kondratieff and Kirchner 1984, their Fig. 6) appear indistinguishable as such: the distal anterior tip is slightly bifurcate, a small ventral subterminal knob is present, and the subterminal forked structure includes paired, ventrally-directed triangular processes and paired somewhat dorsally-directed subtruncate processes. Females from southern Illinois were indistinguishable from those from the Connecticut and Essex Co., Virginia (Fig. 24) localities noted above.

### Prostoia ozarkensis

Baumann & Grubbs sp. n.

http://zoobank.org/8DE0A193-C546-46EB-A70E-DF8FEAFEBA33

http://species-id.net/wiki/Prostoia_ozarkensis

http://lsid.speciesfile.org/urn:lsid:Plecoptera.speciesfile.org:TaxonName:463936

Figs 25–32 , 42

#### Description.

**Figures 25–32.**
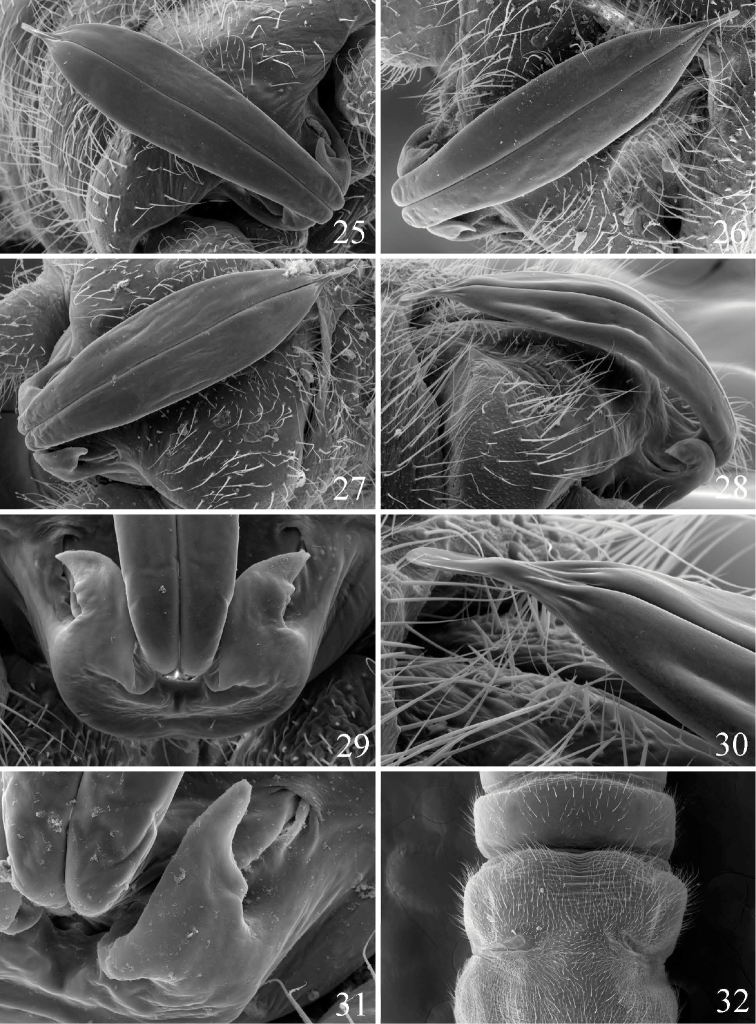
*Prostoia ozarkensis* sp. n., scanning electron micrographs, **25** USA, Arkansas, Buffalo River, male, epiproct, dorsal view **26** USA, Missouri, Bryant Creek, male, epiproct, dorsal view **27** USA, Illinois, Hutchins Creek, male, epiproct, dorsal view **28** USA, Oklahoma, Baron Creek, male, epiproct tip, lateral view **29** USA, Oklahoma, Baron Creek, male, epiproct base, dorsal view **30** USA, Oklahoma, Baron Creek, male, epiproct tip, lateral view **31** USA, Illinois, Hutchins Creek, male, epiproct base, dorsal view **32** USA, Oklahoma, Baron Creek, female, abdominal terminalia, ventral view.

**Male.** Macropterous. Forewing length 7.0–8.0 mm; body length 6.0–6.5 mm. Wings mottled with light band in forewing beyond cord. General body color brown. Epiproct ventral sclerite recurved over abdomen, gradually widening anterior to base, widest in distal third, narrowing mark to an acute, parallel-sided tip, rounded apically (Figs 25–30); recurved portion of ventral sclerite deflected downward at approximately the midpoint (Fig. 28). Paraprocts broadest basally, extending beyond base of ventral sclerite, subquadrate for ca. 2/3 length, with a triangular distal portion that is slightly flared laterally (Figs 29, 31). Vesicle present. Hypoproct sclerotized, broad at base, tapering markedly to a rounded, narrow apex.

**Female.** Macropterous. Forewing length 8.0–9.0 mm; body length 7.0–7.5 mm. Wing and body coloration similar to male. Seventh and eighth abdominal sterna fused medially, subgenital plate of eighth sternum scarcely extending over anterior portion of ninth sternum, barely concave medially with slightly rounded lateral lobes (Fig. 32).

**Nymph.** Undescribed.

#### Material examined

(also provided in Suppl. material 1). Holotype ♂, in 75% ethyl alcohol, **USA**, **Missouri**, Webster County, Bryant Creek, Hwy 14, 3 mi SW Evans, 36.8870, -92.4733, 22 February 1972, R.W. Baumann & S.W. Szczytko, (USNM). Paratypes: **Arkansas**, Benton Co., White River, 21 February 1943, W. Larimore, ♂ (INHS); White River, Rogers, 6 March 1943, W. Larimore, ♂, 2♀ (INHS); Carroll Co., Big Clifty Creek, SW ¼ Sec 4 T19N R27W, 8 March 1963, O. Hite & L.R. Aggus, 2♀ (INHS); Osage Creek, Hwy. 60, Osage, 36.1887, -93.4152, 16 March 1963, O. Hite & L. Aggus, ♀ (INHS); Madison Co., War Eagle, Hwy 16 & Hwy 45, 36.2020, -93.8569, 10 March 1962, L.O. Warren, 9♂, 11♀ (BYUC); Kings River, Hwy 21, 4 mi N Kingston, 36.0882, -93.5417, 8 March 1963, O. Hite & M. Wall, ♀ (INHS); Marion Co., Crooked Creek, Hwy. 62, 36.2458, -92.8348, 16 March 1963, O. Hite & L. Aggus, ♀ (INHS); Georges Creek, Hwy. 62, 36.2533, -92.7487, 16 March 1963, O. Hite & L. Aggus, ♂ (INHS); Newton Co., Add Creek, Hwy 43, Ponca, 36.0210, -93.3600, 25 March 1972, R.W. Baumann & S.W. Szczytko, ♂, 4♀ (BYUC); Buffalo River, Hwy 74, near Ponca, 36.0221, -93.3547, 25 March 1972, R.W. Baumann, ♀ (BYUC); same site, 8 February 1976, McCraw & Kittle, 3♂, ♀ (BYUC); Buffalo River, Boxley, 35.9610, -93.4040, 16 March 2002, B.C. Kondratieff & R. Zuellig, 2♂ (CSUC); Searcy Co., Big Creek, St. Rd. 14, 35.9789, -92.4815, 17 March 1963, O. Hite & L. Aggus, 4♀ (INHS); Stone Co., Wallace Creek, State Rd. 14, 35.7764, -91.8872, 17 March 1963, O. Hite & L. Aggus, ♀ (INHS); Sylamore Creek, St. Rd. 9, 35.9419, -92.1201, 17 March 1963, O. Hite & L. Aggus, ♂ (INHS); Rocky Bayou, State Rd. 14, 35.8598, -92.0469, 17 March 1963, O. Hite & L. Aggus, 2♂, 4♀ (INHS); Van Buren Co., Peyton Creek, Hwy. 65, 35.7881, -92.5397, 17 March 1963, O. Hite & L. Aggus, ♀ (INHS); Washington Co., War Eagle Creek, SW ¼ Sec 19 T18N R28W, 28 February 1963, O. Hite & L.R. Aggus, ♀ (INHS); same but 14 March 1963, O. Hite & L.R. Aggus, ♂ (INHS); no locality data, 20 March 1962, O. Hite & M. Hite, ♂, 2♀ (INHS). **Illinois**, Union Co., Hutchins Creek, Wolf Lake, 37.5107, -89.3773, 13 March 1946, H.H. Ross & B.D. Burks, ♂, ♀ (INHS); Hutchins Creek, 5.4 km E Wolf Lake, 93-152, T11S, R2W, S31, 37.5107, -89.3776, 19 April 1993, M.A. Harris & D.W. Webb, ♂, ♀ (INHS). **Missouri**, Bollinger Co., Whitewater River, Alliance, 37.5791, -90.0013, 6 March 1958, Ross & Stannard, ♂ (INHS); Christian Co., Bull Creek, Hwy W, 3 March 1972, R.W. Baumann & B.K. Newman, 3♂, 5♀ (BYUC); same site, 20 March 1972, B.K. Newman, ♀ (BYUC); Crawford Co., Meramec River, N Steeleville at MO 19, 37.9889, -91.3761, 4 February 2012, R.E. DeWalt & S.K. Ferguson, 2♂, ♀, 5 nymphs (INHS); Meramec River, Steeleville, 37.9849, -91.3724, 6 March 1958, Ross & Stannard, ♂, 3♀ (INHS); Huzzah Creek, Dilliard, Mark Twain [Clark] National Forest, 37.7406, -91.2029, 6 March 1958, Ross & Stannard, ♂, ♀ (INHS); Dade Co., Turnback Creek, Hwy O, E Greenfield, 37.4023, -93.8020, 19 February 1972, D.A. Boehne, 2♂, 4♀ (BYUC); same site, 18 March 1972, D.A. Boehne, ♂, ♀ (BYUC); Douglas Co., Bryant Creek, Hwy 14, 3 mi SW Evans, 36.8870, -92.4733, 22 February 1972, R.W. Baumann & S.W. Szczytko, 12♂, 25♀ (BYUC); Bryant Creek, Hwy 14, near Gentryville, 36.8868, -92.4734, 14 March 1972, R.W. Baumann & C.D. Inman, ♂, 9♀ (BYUC); Bryant Creek, Gentryville, 18 February 1962, Ross & Stannard, ♂, 4♀ (INHS); North Fork White River, Hwy 14, Twin Bridges, 36.8109, -92.1492, 22 February 1972, R.W. Baumann, ♂, 2♀ (BYUC); Franklin Co., Indian Creek, 1.5 mi S Piney park at Hwy K, 38.2692, -90.9447, 4 February 2012, R.E. DeWalt & S.K. Ferguson, 11♂, 5♀, 8 nymphs (INHS); Greene Co., Little Pomme de Terre River, Hwy 65, near Fair Grove, 37.4161, -93.1452, 15 February 1972, R.W. Baumann, 2♂ (BYUC); same site, 24 March 1972, R.W. Baumann, ♀ (BYUC); Lawrence Co., White Oak Creek, near Red Oak, 37.2291, -94.0276, 19 March 1972, R.W. Baumann, ♀ (BYUC); Shannon Co., Current River, Hwy B, Cedar Grove, 37.4189, -91.6029, 17 March 2002, B.C. Kondratieff & R. Zuellig, ♂ (CSUC); Jacks Fork River, Hwy S, Creek, 17 March 2002, B.C. Kondratieff & R. Zuellig, 5♂, 6♀ (CSUC); Manan Creek, Hwy 106, W Eminence, 37.1461, -91.3792, 16 March 2002, B.C. Kondratieff & R. Zuellig, ♂ (CSUC); Big Shawnee Creek, 2 mi E Eminence at MO 106, 37.1528, -91.3131, 5 February 2012, R.E. DeWalt & S.K. Ferguson, ♂ (INHS), Shawnee Creek, Hwy 106, N Winona, 37.1528, -91.3132, 17 March 2002, B.C. Kondratieff & R. Zuellig, 3♂, 4♀ (CSUC); Taney Co., Bull Creek, Hwy 76, 36.7311, -93.1933, 28 February 1972, B.K. Newman, ♂, 3♀ (BYUC); same site, 8 March 1972, B. K. Newman, 4♂, 3♀ (BYUC); Texas Co., Big Piney River, Hwy RA, N Simmons, 17 March 2002, B.C. Kondratieff & R. Zuellig, ♀ (CSUC); Hog Creek, S Houston, 37.2400, -91.9527, 17 March 2002, B.C. Kondratieff & R. Zuellig, 2♀ (CSUC); Jacks Fork River, 5 mi S Pine Crest, 37.0563, -91.6679, 17 February 1962, Ross & Stannard, ♂ (INHS); Wright Co., Gasconade River, Hwy E, 9 mi. NE Hartville, 37.3135, -92.3988, 13 March 1987, B.C. Poulton, 2♀ (BYUC). **Oklahoma**, Adair Co., Ballard Creek, 36.0924, -94.5881, 20 February 1972, B.P. Stark, ♂, 4♀ (BYUC); unnamed stream, Hwy 59, Baron, 35.9195, -94.6199, 20 February 1972, B.P. Stark, 2♂, 2♀ (BYUC); Delaware Co., Flint Creek, 36.1942, -94.7069, 19 February 1984, B.C. Poulton, 2♂, 2♀ (BYUC).

#### Etymology.

The specific epithet recognizes that this species is broadly widespread across the Ozark Plateau region of southern Missouri, northern Arkansas, and northeastern Oklahoma, with one additional isolated locality in southwestern Illinois. The common name Ozark Forestfly is proposed for this species (Stark et al. 2012).

#### Diagnosis.

*Prostoia besametsa*, *Prostoia completa*, and *Prostoia ozarkensis* sp. n. appear to form a closely-related species group based primarily on structural similarities of the short, compact lateral arms of the male dorsal sclerite and the female 8^th^ sternum that bears a faint medial notch with poorly-developed lateral lobes. The combination of the narrow, v-shaped epiproct tip and the western Nearctic distribution of *Prostoia besametsa* easily separates this species from *Prostoia completa* and *Prostoia ozarkensis* sp. n. The epiproct of *Prostoia completa* and *Prostoia ozarkensis* sp. n. narrow markedly to an acute, parallel-sided tip. In addition, the lateral arms of *Prostoia besametsa* do not extend past the epiproct base whereas in *Prostoia completa* and *Prostoia ozarkensis* sp. n. the lateral arms are noticeably longer. *Prostoia ozarkensis* sp. n. closely resembles *Prostoia completa* in both the male and female adult stages. Whereas females of the two species appear indistinguishable, males can be separated by close examination of details of the lateral arms and the overall shape of the ventral sclerite. The lateral arms of *Prostoia ozarkensis* sp. n. are short, pointed apically, and bear a stout nub on the outer surface (Fig. 29). While in *Prostoia completa* the lateral arm is longer, scythe-shaped, and has a smooth outer margin (Fig. 15). The ventral sclerite of *Prostoia ozarkensis* sp. n. is recurved in lateral aspect, especially along the ventral margin (Fig. 28) and expanded dorsally toward the apex (Fig. 25). Conversely, in *Prostoia completa* the ventral sclerite is nearly straight in lateral aspect (Fig. 11) and narrow and nearly parallel-sided dorsally (Figs 9, 10).

*Prostoia ozarkensis* sp. n. overlaps in range only with *Prostoia similis* (Fig. 42), but the combination of the long, sinuate lateral arms and the well-developed lateral lobes of the female 8^th^ sternum easily distinguish the latter species from each of the four other *Prostoia* species. With the surprising discovery of *Prostoia hallasi* from southern Illinois, the distribution of this species is likely far from understood and there is no reason to preclude its presence west of the Mississippi River into the Interior Highland region. The ornate epiproct tip and absence of lateral arms of *Prostoia hallasi* are distinctive features that make it easy to identify males of this species. Additionally, *Prostoia hallasi* is the only *Prostoia* species with a convex subgenital plate.

#### Remarks.

*Prostoia ozarkensis* sp. n. specimens from the Ozark Plateau, including the Boston Mountains, consistently exhibit distinctive male characters that set it apart from widespread *Prostoia completa*. Specimens from states to the east, namely Indiana and Kentucky, are more difficult to separate consistently and even show variability in the same population. The *Prostoia completa* records presented in Poulton and Stewart (1991) likely now pertain to *Prostoia ozarkensis* sp. n., but very few of their specimens were available for study. The same also applies for *Prostoia completa* reported in Stark and Stewart (1973), Ernst et al. (1984), Ernst and Stewart (1985a, 1985b, 1986), Jop and Stewart (1987), Phillips and Kilambi (1994), and Harp and Robison (2006).

### Prostoia similis

(Hagen)

http://species-id.net/wiki/Prostoia_similis

http://lsid.speciesfile.org/urn:lsid:Plecoptera.speciesfile.org:TaxonName:6094

Figs 33–40 , 42

Taeniopteryx similis Hagen, 1861: 34. Holotype ♂ (USNM), Washington D.C., USA.
Nemoura similis : Banks 1907: 14.
Nemoura divergens : Claassen 1923: 282. Syn. Illies, 1966: 221.
Nemoura divergens : Needham and Claassen 1925: 203.
Nemoura similis : Needham and Claassen 1925: 214.
Nemoura (Prostoia) similis : Ricker 1952: 49.
Prostoia similis : Illies 1966: 221.
Prostoia similis : Zwick 1973: 346.
Prostoia similis : Baumann 1975: 27.
Prostoia similis : Poulton and Stewart 1991: 30.

#### Material examined

(Suppl. material 1).

#### Distribution.

Canada: ON (New provincial record), PQ (DeWalt et al. 2013); USA: CT, DE, IL, IN, KY, MA, MD, ME, MI, MN, MO, NY, OH, PA, SC, VA, WI, WV (DeWalt et al. 2013), TN (New state record).

#### Remarks.

*Prostoia similis* is readily distinguished from all other *Prostoia* species by the dorsal sclerite of the epiproct bearing long and sinuate lateral arms. As stated earlier, the ranges of *Prostoia completa* and *Prostoia similis* overlap extensively. Examination of large collections of *Prostoia similis* and *Prostoia completa* from the Great Lakes region has revealed that the former species appears to be markedly less common with increasing latitude (Grubbs et al. 2012). In comparison to *Prostoia completa*, there are far fewer historical (pre-1960) and contemporaneous collections of *Prostoia similis* from Michigan, Minnesota, Wisconson, and Ontario.

**Figures 33–40.**
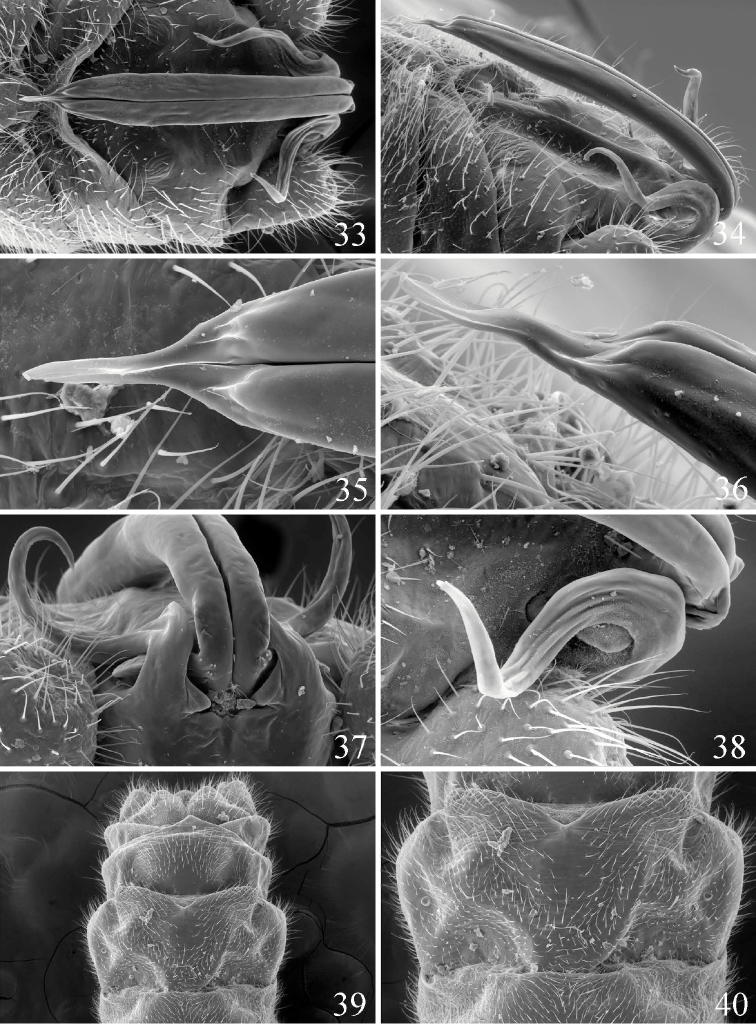
*Prostoia similis*, scanning electron micrographs, **33** USA, Virginia, Big Run, male, epiproct, dorsal view **34** USA, Virginia, McClure River, male, epiproct, lateral view **35** USA, Virginia, McClure River, male, epiproct tip, dorsal view **36** USA, Virginia, McClure River, male, epiproct tip, lateral view **37** USA, Virginia, McClure River, male, epiproct base, caudal view **38** USA, Virginia, Big Run, male, epiproct base, dorsal view **39** USA, McClure River, Virginia, female, abdominal terminalia, ventral view **40** USA, Virginia, McClure River, female, abdominal terminalia, ventral view.

**Figure 41.**
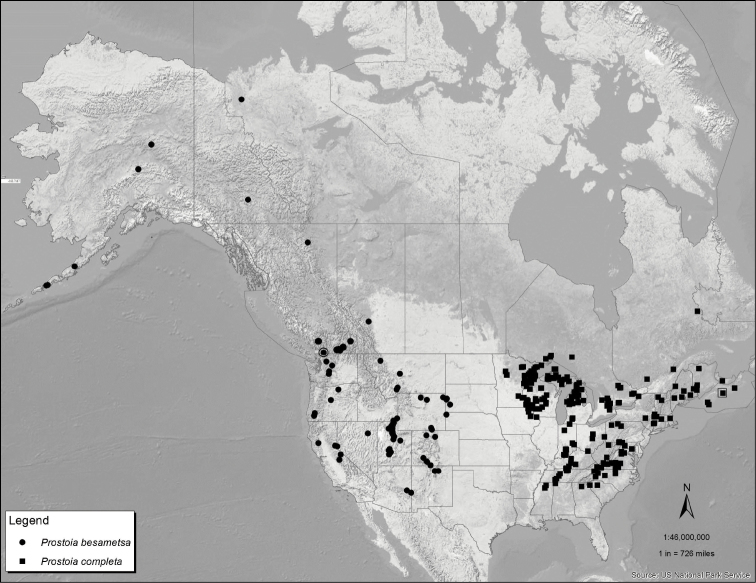
Distribution map for *Prostoia besametsa* (circles) and *Prostoia completa* (squares). The open symbols enclosing the solid symbols refer to the type localities for the two species.

**Figure 42.**
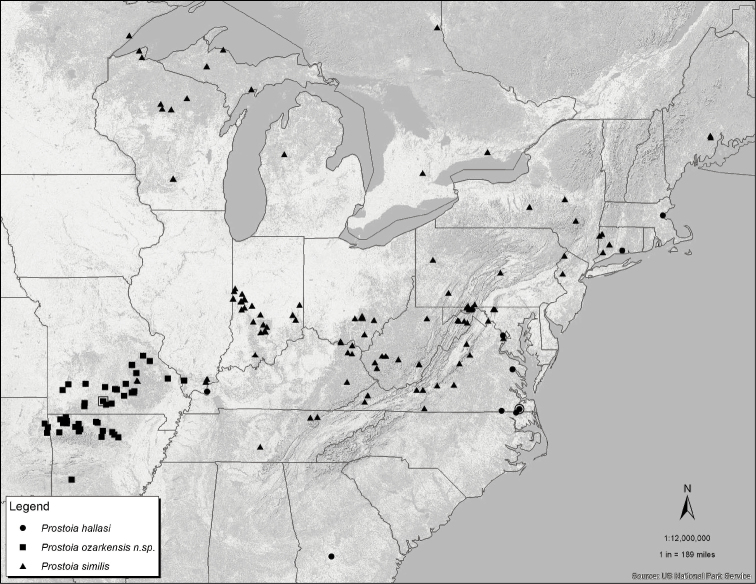
Distribution map for *Prostoia hallasi* (circles), *Prostoia ozarkensis* sp. n. (squares), and *Prostoia similis* (triangles). The open symbols enclosing the solid symbols refer to the type localities for the three species.

## Supplementary Material

XML Treatment for Prostoia besametsa

XML Treatment for Prostoia completa

XML Treatment for Prostoia hallasi

XML Treatment for Prostoia ozarkensis

XML Treatment for Prostoia similis

## Supplementary material

Supplementary material 1 Raw locality data for all *Prostoia* material examined in this study. doi: 10.3897/zookeys.401.7299.app1 Data type: MS Excel spreadsheet (xls) of species locality data. Explanation note: Supplementary material 1 is an MS Excel spreadsheet with all specimen locality data used in this article. This dataset is made available under the Open Database License (http://opendatacommons.org/licenses/odbl/1.0/). The Open Database License (ODbL) is a license agreement intended to allow users to freely share, modify, and use this Dataset while maintaining this same freedom for others, provided that the original source and author(s) are credited.Scott A. Grubbs, Richard W. Baumann, R. Edward DeWalt, Tari Tweddale
